# Changes in Serological Immunology Measures in UK and Kenyan Adults Post-controlled Human Malaria Infection

**DOI:** 10.3389/fmicb.2016.01604

**Published:** 2016-10-13

**Authors:** Susanne H. Hodgson, David Llewellyn, Sarah E. Silk, Kathryn H. Milne, Sean C. Elias, Kazutoyo Miura, Gathoni Kamuyu, Elizabeth A. Juma, Charles Magiri, Alfred Muia, Jing Jin, Alexandra J. Spencer, Rhea J. Longley, Thomas Mercier, Laurent Decosterd, Carole A. Long, Faith H. Osier, Stephen L. Hoffman, Bernhards Ogutu, Adrian V. S. Hill, Kevin Marsh, Simon J. Draper

**Affiliations:** ^1^The Jenner Institute, University of OxfordOxford, UK; ^2^Laboratory of Malaria and Vector Research, NIH-National Institute of Allergy and Infectious DiseasesRockville, MD, USA; ^3^Centre for Geographical Medical Research (Coast), Kenya Medical Research Institute—Wellcome TrustKilifi, Kenya; ^4^Centre for Clinical Research, Kenya Medical Research InstituteNairobi, Kenya; ^5^Centre for Research in Therapeutic Sciences, Strathmore UniversityNairobi, Kenya; ^6^Division of Clinical Pharmacology, Hôpital Beaumont, Université de LausanneLausanne, Switzerland; ^7^Sanaria Inc.Rockville, MD, USA; ^8^Department of Tropical Medicine, University of OxfordOxford, UK

**Keywords:** malaria, challenge, falciparum, immunity, CHMI, ELISA, GIA, ADRB

## Abstract

**Background:** The timing of infection is closely determined in controlled human malaria infection (CHMI) studies, and as such they provide a unique opportunity to dissect changes in immunological responses before and after a single infection. The first Kenyan Challenge Study (KCS) (Pan African Clinical Trial Registry: PACTR20121100033272) was performed in 2013 with the aim of establishing the CHMI model in Kenya. This study used aseptic, cryopreserved, attenuated *Plasmodium falciparum* sporozoites administered by needle and syringe (PfSPZ Challenge) and was the first to evaluate parasite dynamics post-CHMI in individuals with varying degrees of prior exposure to malaria.

**Methods:** We describe detailed serological and functional immunological responses pre- and post-CHMI for participants in the KCS and compare these with those from malaria-naïve UK volunteers who also underwent CHMI (VAC049) (ClinicalTrials.gov NCT01465048) using PfSPZ Challenge. We assessed antibody responses to three key blood-stage merozoite antigens [merozoite surface protein 1 (MSP1), apical membrane protein 1 (AMA1), and reticulocyte-binding protein homolog 5 (RH5)] and functional activity using two candidate measures of anti-merozoite immunity; the growth inhibition activity (GIA) assay and the antibody-dependent respiratory burst activity (ADRB) assay.

**Results:**Clear serological differences were observed pre- and post-CHMI by ELISA between malaria-naïve UK volunteers in VAC049, and Kenyan volunteers who had prior malaria exposure. Antibodies to AMA1 and schizont extract correlated with parasite multiplication rate (PMR) post-CHMI in KCS. Serum from volunteer 110 in KCS, who demonstrated a dramatically reduced PMR *in vivo*, had no *in vitro* GIA prior to CHMI but the highest level of ADRB activity. A significant difference in ADRB activity was seen between KCS volunteers with minimal and definite prior exposure to malaria and significant increases were seen in ADRB activity post-CHMI in Kenyan volunteers. Quinine and atovaquone/proguanil, previously assumed to be removed by IgG purification, were identified as likely giving rise to aberrantly high *in vitro* GIA results.

**Conclusions:** The ADRB activity assay is a promising functional assay that warrants further investigation as a measure of prior exposure to malaria and predictor of control of parasite growth. The CHMI model can be used to evaluate potential measures of naturally-acquired immunity to malaria.

## Introduction

Although encouraging evidence suggests that the epidemiology of *Plasmodium falciparum* malaria is changing across certain parts of Africa (Okiro et al., 2007), the burden of disease from malaria remains a major public health problem, with approximately 214 million cases and 438,000 deaths worldwide in 2015 (WHO, 2015). Despite considerable efforts, the development of a highly effective vaccine against malaria infection, disease, or transmission remains elusive (Halbroth and Draper, 2015).

Controlled human malaria infection (CHMI) studies have become a vital, routine tool to accelerate vaccine and drug development against *P. falciparum* (McCarthy et al., 2011; Sauerwein et al., 2011; Duncan and Draper, 2012; Roestenberg et al., 2012). By infecting healthy volunteers with *P. falciparum* parasites in a controlled environment, CHMI studies have been used to deselect vaccine candidates to ensure only the most promising move forward to evaluation in field studies (Sheehy et al., 2013a).

Whilst routinely performed in American, European, and Australian centers with malaria-naïve subjects, modern *P. falciparum* CHMI studies have rarely been performed in malaria-endemic regions or involved volunteers with prior exposure to malaria (Sauerwein et al., 2011; Sheehy et al., 2013a). This has primarily been due to the lack of access to appropriate facilities to perform mosquito-bite CHMI trials in malaria endemic countries (Sheehy et al., 2013a). The development of aseptic, cryopreserved *P. falciparum* sporozoites (NF54 strain) for injection (PfSPZ Challenge) by the biotechnology company Sanaria Inc., has helped overcome this problem (Epstein, 2013; Roestenberg et al., 2013; Sheehy et al., 2013b; Gómez-Pérez et al., 2015; Lyke et al., 2015) and recently, CHMI studies have been undertaken in Tanzania, Kenya and Mali using PfSPZ Challenge (Hodgson et al., 2014; Shekalaghe et al., 2014).

Given the timing of infection is closely controlled in CHMI studies, they provide the opportunity to dissect in detail changes in immunological responses before and after a single infection. In malaria-exposed individuals, they also provide the opportunity to assess the effect of prior exposure to *P. falciparum*, and by inference, naturally-acquired immunity (NAI) on parasite growth dynamics.

The Kenyan Challenge Study (KCS) was the first modern CHMI study performed in Kenya (Hodgson et al., 2014). In this study, all 28 volunteers were successfully infected with malaria and one participant (volunteer 110) remained undiagnosed by thick-film microscopy 21 days post-injection of PfSPZ Challenge (C+21). Parasite multiplication rate (PMR), a measure of the fold change in blood-stage parasitaemia over 48 h, was calculated for each participant and a range of PMRs were seen in diagnosed volunteers (median 11.1, range 5.2–18), whilst volunteer 110 had a markedly reduced PMR of 1.3.

KCS was the first CHMI study to attempt to define the degree of prior exposure (and therefore NAI) to malaria prior to CHMI. Whilst the enrolled volunteers were concluded to have low to moderate NAI, the range of PMRs seen and the finding of one individual clearly capable of controlling blood-stage parasite growth *in vivo* suggested PMR as a potential measure of NAI against which immunological assays, including functional assays, could be assessed.

Here were describe detailed serological and functional immunological responses for participants in KCS before and after CHMI and compare these with those from malaria-naïve UK volunteers who also underwent CHMI using PfSPZ Challenge (in a previously reported clinical trial called VAC049) (Sheehy et al., 2013b). Of the many potential blood-stage antigens, we chose to assess antibody responses to three well-known anti-merozoite vaccine candidates; merozoite surface protein 1 (MSP1), apical membrane protein 1 (AMA1), and reticulocyte-binding protein homolog 5 (RH5) (Sheehy et al., 2012b; Biswas et al., 2014; Douglas et al., 2015; Halbroth and Draper, 2015; Payne et al., 2016). We also chose to assess two candidate measures of anti-merozoite immunity; the widely used growth inhibition activity (GIA) assay, which assesses the ability of purified IgG to inhibit *P. falciparum* growth *in vitro* in a cell-independent manner (Malkin et al., 2005; Duncan et al., 2012), and the antibody-dependent respiratory burst activity (ADRB) assay which assesses the ability of antibody to opsonize merozoites and induce the release of reactive oxygen species (ROS) from polymorphonuclear neutrophils *in vitro* (Joos et al., 2010; Kapelski et al., 2014; Llewellyn et al., 2014, 2015).

This work is the first to compare changes in key immunological measures pre- and post-acute *P. falciparum* infection in adults with varying degrees of prior exposure to malaria. As such, these findings provide important information on the value of immunological measures at predicting prior exposure and therefore immunity to *P. falciparum*.

## Materials and methods (see Supplementary Information)

### Controlled human malaria infection studies

CHMI was undertaken by needle and syringe administration of aseptic, un-attenuated *P. falciparum* sporozoites, cryopreserved at known concentrations and stored in liquid phase liquid nitrogen (PfSPZ Challenge) (Sheehy et al., 2013b; Hodgson et al., 2014).

**Figure 1:**
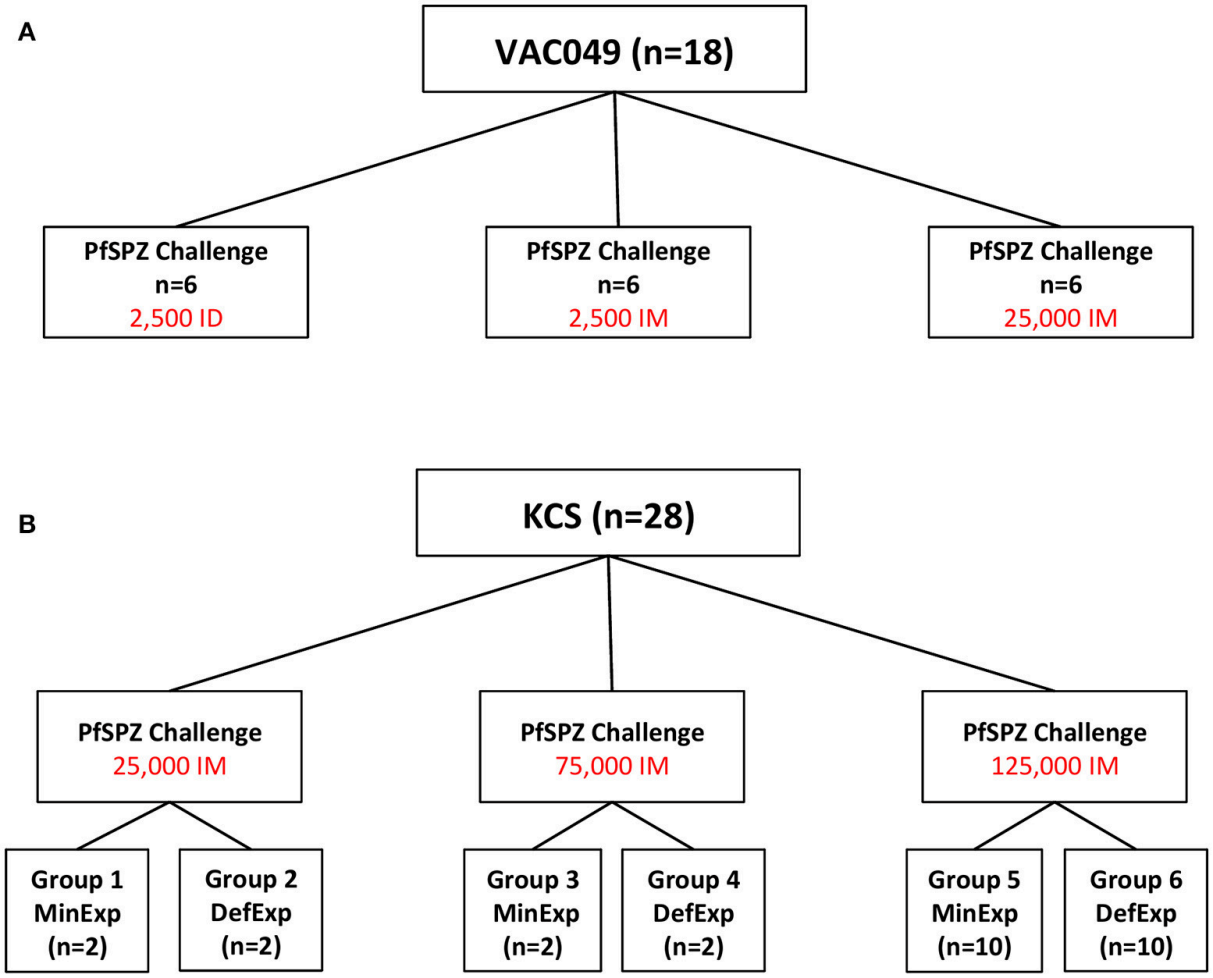
**Design of Studies. (A)** VAC049 was a UK CHMI study of PfSPZ Challenge administered to malaria-naïve, UK volunteers. **(B)** KCS was a Kenyan Challenge Study of PfSPZ Challenge administered to Kenyan volunteers. In each study in each group, the total dose of sporozoites was split between two injection sites and administered as two 50 μL injections, one in each deltoid. ID, intradermal; IM, intramuscular; MinExp, minimal prior exposure to malaria; DefExp, definite prior exposure to malaria.

KCS was an open label, randomized pilot study with blinded laboratory outcome assessment, evaluating PfSPZ Challenge administered intramuscularly (IM) to 28 individuals with varying degrees of prior exposure to *P. falciparum* (Figure 1) (Hodgson et al., 2014). A dose escalation study design was applied to allow assessment of safety prior to administration of the target dose of 125,000 sporozoites. Volunteers in KCS were grouped into those with minimal (MinExp) or definite (DefExp) prior exposure to malaria according to antibody responses to schizont extract and merozoite surface protein 2 (MSP2) as previously described (Hodgson et al., 2014, 2015). The study was conducted at the Kenya Medical Research Institute (KEMRI) Centre for Clinical Research, Nairobi, Kenya, and registered with the Pan African Clinical Trial Registry (PACTR20121100033272).

VAC049 was an open label, non randomized pilot study with blinded laboratory outcome assessment evaluating the safety and infectivity of various doses of PfSPZ Challenge administered IM and intradermally (ID) in 18 malaria-naïve, UK adults (Figure 1) (Sheehy et al., 2013b). The study was conducted at the Centre for Clinical Vaccinology and Tropical Medicine, University of Oxford, Oxford, UK and registered with ClinicalTrials.gov (NCT01465048).

Both studies were conducted according to the principles of the Declaration of Helsinki and in accordance with Good Clinical Practice (GCP). In both studies, participants were treated with a 3-day curative course of Atovaquanone/Proguanil at C+21.

### ELISAs

All ELISAs were standardized and performed according to published protocols using serum (Miura et al., 2008; Sheehy et al., 2012a,b; see Supplementary Information). Anti-AMA1 (full-length), anti-MSP1 (19 kDa C−terminal region, MSP1_19_), and anti-RH5 (full-length) ELISAs were performed at the Jenner Institute, University of Oxford whilst the anti-schizont ELISA was performed at KEMRI-Wellcome Trust, Centre for Geographical Medical Research (Coast), Kilifi. Due to limited sample volumes, antibodies to schizont extract were not assessed for subjects enrolled in VAC049. Antibody unit = the dilution of the sample that would give an OD of 1.0 under the given ELISA conditions.

### Growth inhibition activity assay

The ability of antibodies to inhibit growth of *P. falciparum* 3D7 clone parasites *in vitro* was assessed by a standardized GIA assay using purified IgG at 10 mg/mL as previously described (Malkin et al., 2005). This assay was performed at the GIA Reference Center (Laboratory of Malaria and Vector Research, NIH). The 3D7 clone was originally isolated from the NF54 parental strain and is thus closely related to the PfSPZ Challenge inoculum.

### Antibody-dependent respiratory burst activity assay

The ability of donor neutrophils to produce reactive oxygen species (ROS) in the presence of test serum and *P. falciparum* 3D7 clone merozoites was assessed using a standardized assay at the Jenner Institute, University of Oxford (Llewellyn et al., 2015). 100 μL serum diluted 1:50 in PBS added to 50 μL of isolated human PMNs at 1 × 10^7^ PMNs/mL was tested against 3D7 parasites (see Supplementary Information).

### Parasite growth modeling

Sampling for qPCR to measure blood-stage parasitemia was performed 1–2 times a day and qPCR conducted as previously described (Sheehy et al., 2012b). Results were modeled using simple linear regression (Douglas et al., 2013; Hodgson et al., 2014) to estimate PMR (see Supplementary Information). PMR is the fold change in number of parasites in the blood over one lifecycle (48 h). Liver to blood inoculum (LBI) is the total number of parasites released from the liver at C+6.5.

### Statistical analysis

The study was designed to assess proof of concept and group sizes were pragmatic rather than based on a formal sample size calculation for any one defined endpoint; statistical analyses were therefore primarily descriptive in nature and results interpreted with caution. Multiple analyses to interrogate the relationship between outcome measures were hypothesis generating and recognized to require replication in future studies.

Results were compared between groups using the Mann-Whitney U test, Wilcoxin matched-pairs signed rank test or Kruskal-Wallis test as appropriate. Correlations were assessed using Spearman's rank correlation coefficient. Data were analyzed using GraphPad Prism version 5.03 for Windows (GraphPad Software Inc., USA).

## Results

### Infectivity of CHMI studies

In VAC049, 14 volunteers were successfully infected, with four volunteers qPCR negative throughout follow-up (Figures S1A,C; Sheehy et al., 2013b). In KCS, all 28 volunteers were successfully infected as assessed by qPCR (Figures S1B,C), however, one volunteer (110) was undiagnosed by thick film microscopy by C+21 with a notably reduced PMR compared to the other volunteers (Figure S2; Hodgson et al., 2014).

There was no significant difference in parasitaemia at diagnosis (p/mL measured by qPCR) between volunteers diagnosed in VAC049 and KCS (*p* = *0.213, Mann-Whitney U test*, data not shown). However, a significant difference in time to diagnosis was seen between studies (*p* = *0.006, Log rank test*; Figure S1C), most likely reflecting variation in LBI due to differences in number and route of administration of sporozoites between studies (Figure S3, Figure 1).

### Analyses of antibody responses

Antibody responses in VAC049 to MSP1_19_ and AMA1 in volunteers infected with malaria were significantly induced following a single infection (Figure S4A) and were comparable to those reported in UK controls following mosquito-bite CHMI (Biswas et al., 2014). Responses to RH5 were not detected in UK adults post-CHMI, in agreement with this antigen being weakly recognized in the context of natural malaria infection (Douglas et al., 2011; Villasis et al., 2012; Tran et al., 2014).

Comparison of ELISA results between VAC049 and the MinExp volunteers in KCS showed no significant differences at baseline (C−1), consistent with this group of Kenyan volunteers being minimally-exposed and relatively similar to malaria-naïve UK adults (Figure S4B, Table S1). However, at C+35, a significant difference in responses to MSP1_19_ was seen (Figure S4C, Table S1), and a similar trend was observed for AMA1—consistent with some degree of previous B cell priming (Elias et al., 2014). Again, serological responses against RH5 remained largely negative in both groups.

**Figure 2:**
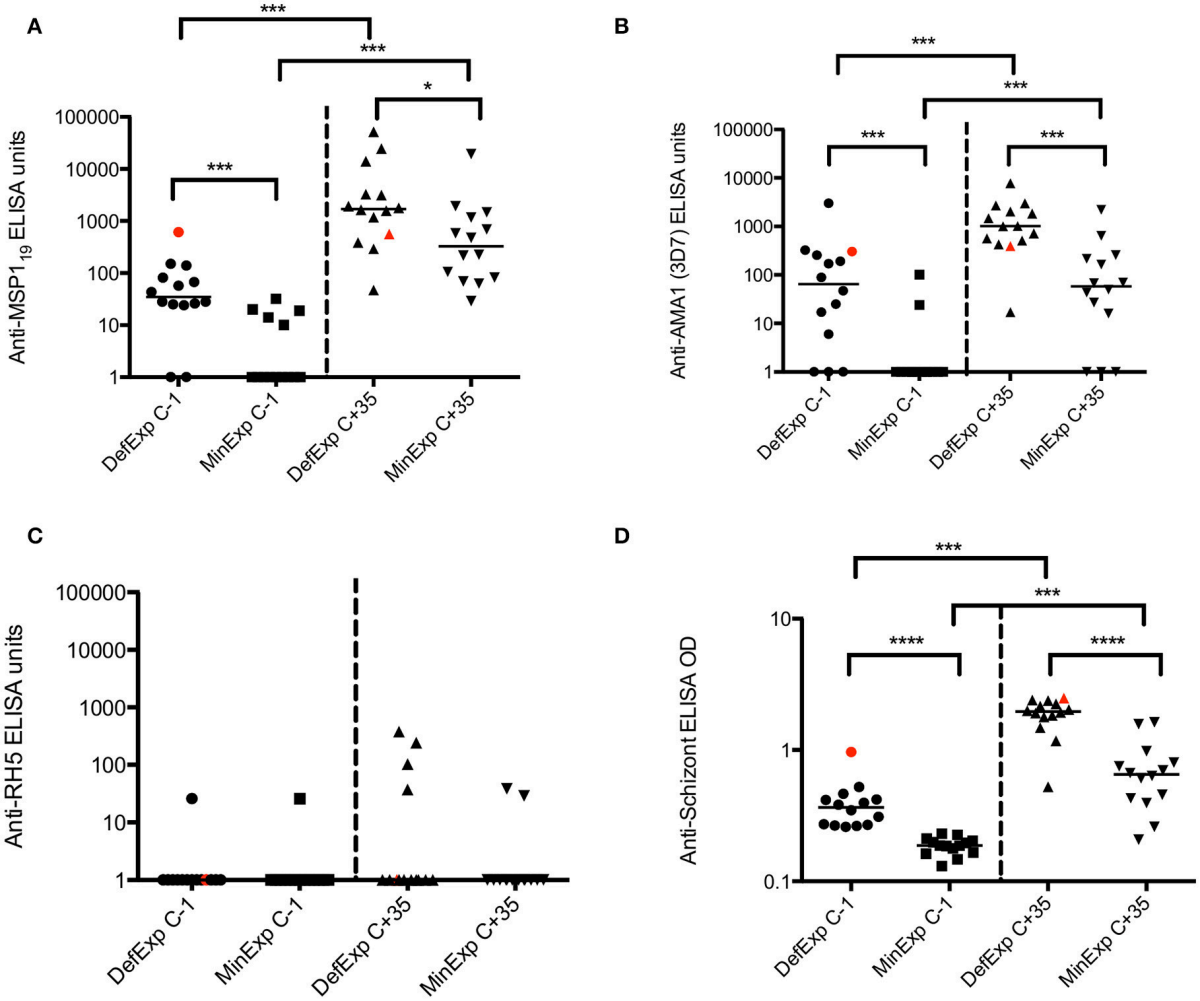
**Serum IgG antibody responses for volunteers with Minimal and Definite prior exposure to malaria in KCS.(A)** MSP1_19_. **(B)** AMA1. **(C)** RH5. **(D)** Schizont extract. Median values are indicated. Mann Whitney U tests and Wilcoxon matched-pairs signed rank tests as appropriate. Data from volunteer 110 highlighted in red. MinExp, minimal prior exposure to malaria; DefExp, definite prior exposure to malaria. C−1, baseline pre-CHMI. C+35 = 35 days post-CHMI. ^*^p < *0.05*, ^***^p < *0.001*, ^****^p < *0.0001*.

In KCS, a significant difference in baseline antibody responses was seen between DefExp and MinExp volunteers for all antigens except RH5 (Figures 2A–D). Consistent with the above, antibody responses to all antigens (with the exception of RH5) were significantly boosted following CHMI for both DefExp and MinExp volunteers (Figures 2A–D) and across all volunteers (Table 1, top row), with the DefExp group showing the highest responses at C+35. Of note, the highest antibody responses to MSP1_19_ and AMA1 in KCS were still lower than those seen following vaccination of UK adults with viral vectored vaccines encoding these antigens (and assessed using the same ELISA assay), but were similar to those recently reported for a cohort of naturally-immune Kenyan adults (Sheehy et al., 2012b; Biswas et al., 2014).

**Table 1:** **Analyses of antibody responses and ADRB activity for KCS**.

**Comparison**	**MSP119 IgG Titre**		**AMA1 IgG Titre**		**RH5 IgG Titre**		**Schizont OD**		**GIA**		**ADRB**	
	***p***	***r***	***p***	***r***	***P***	***r***	***p***	***r***	***p***	***r***	***p***	***r***
C−1v C+35	**<0.0001**		**<0.0001**		0.148		**<0.0001**		**<0.0001**		**<0.0001**	
Parasitemia at diagnosis v C−1	0.042	0.395	0.066	0.359	0.193	0.259	0.202	0.253	0.930	−0.018	0.167	0.274
Parasaemia at diagnosis v C+35	**0.036**	0.405	0.208	0.208	0.960	0.011	**0.037**	0.404	0.283	0.214	0.103	0.321
PMR v C−1	0.886	−0.029	**0.018**	−0.443	0.491	−0.136	**0.044**	−0.384	0.780	−0.337	0.192	−0.254
ADRB C+35 v C+35	**0.0002**	0.649	**0.0001**	0.658	0.111	0.308	**<0.0001**	0.886	0.111	0.308		

n = 28 for all analyses. Correlations were performed using Spearman rank test. Comparisons were performed using Wilcoxon matched-pairs signed rank or Mann Whitney U tests as appropriate (top row only). These analyses were hypothesis driven and key findings require replication in future studies. Results in bold are statistically significant.

For KCS, only antibody responses at C−1 to schizont and AMA1 showed a significant correlation to PMR (*p* = *0.044* and *p* = *0.018* respectively; Table 1). For VAC049, no antibody responses (measured at C+35) were shown to correlate with the number of parasites at diagnosis (Table S1), however, in KCS schizont (C+35) and MSP1_19_ ELISA (C−1 and C+35) associated with parasitemia at diagnosis (Table 1). These data are similar to those previously reported suggesting de novo anti-MSP1_19_ serum IgG responses post-mosquito bite CHMI in UK malaria-naïve control volunteers associate with the duration of blood-stage parasite exposure (Elias et al., 2014).

### Analyses of *in vitro* growth inhibition activity

**Figure 3:**
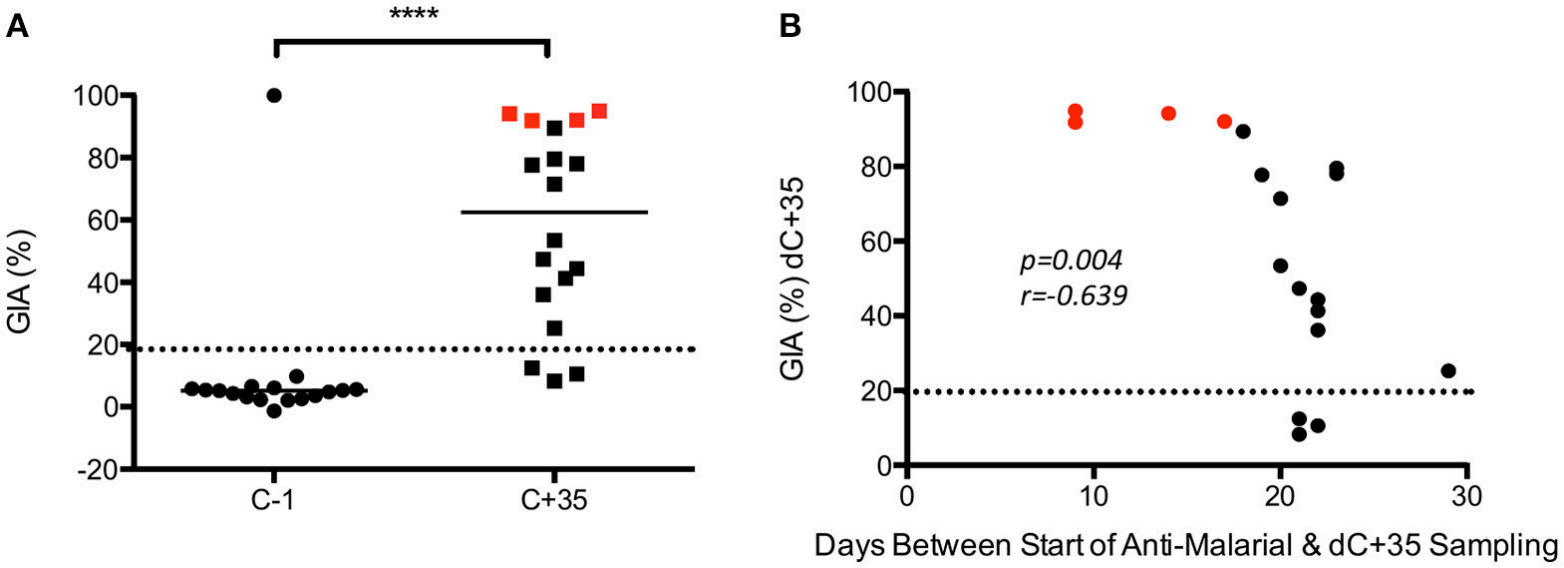
**VAC049 GIA data. (A)** Pre- and post–CHMI. Wilcoxon matched-pairs signed rank test. Individual data and median are shown. **(B)** Correlation between GIA at C+35 and days between start of anti-malarial therapy and sampling at C+35 visit. Spearman rank test. Volunteers not successfully infected are highlighted in red. GIA expected to be < 20% in malaria-naïve individuals (dotted line). C−1 = baseline pre-CHMI. C+35 = 35 days post-CHMI. ^****^p <*0.0001*.

GIA was assessed using purified IgG from serum of VAC049 volunteers before and after CHMI. No result was obtained for volunteer 1224 (Group 1, successfully infected) at C−1 due to a technical failure. A number of unexpected results were seen. Firstly, one subject, (volunteer 1221, group 1, successfully infected) was shown to have GIA of 99.9% at C−1 (expected to be < 20%) (Figure 3A). This volunteer was shown to have a plasma concentration of 11 ng/mL of quinine at C−1 (see supplementary information), confirmed by repeated testing, and likely represented surreptitious quinine use by the volunteer (although the volunteer denied this).

The second finding of note was that GIA increased post-CHMI across the cohort, but most markedly in the four subjects that were not successfully infected in VAC049 (i.e., qPCR negative from C+6.5 until C+21) (Figure 3B). This finding was in contrast to other studies where no GIA was induced in malaria-naïve adults following a single CHMI (Duncan et al., 2011; Biswas et al., 2014). A correlation was seen between GIA at C+35 and days between start of anti-malarial therapy and sampling at C+35. Given that uninfected subjects in VAC049 were treated at C+21, at least 3 days later than the other subjects, they had a shorter interval between completion of their drug therapy and sampling at C+35. It is likely therefore that the increase in GIA seen post-CHMI is secondary to a residual effect from Atovaquone/Proguanil (Malarone™), persisting despite purification of IgG, rather than mediated by antibody induced following a single malaria infection.

**Figure 4:**
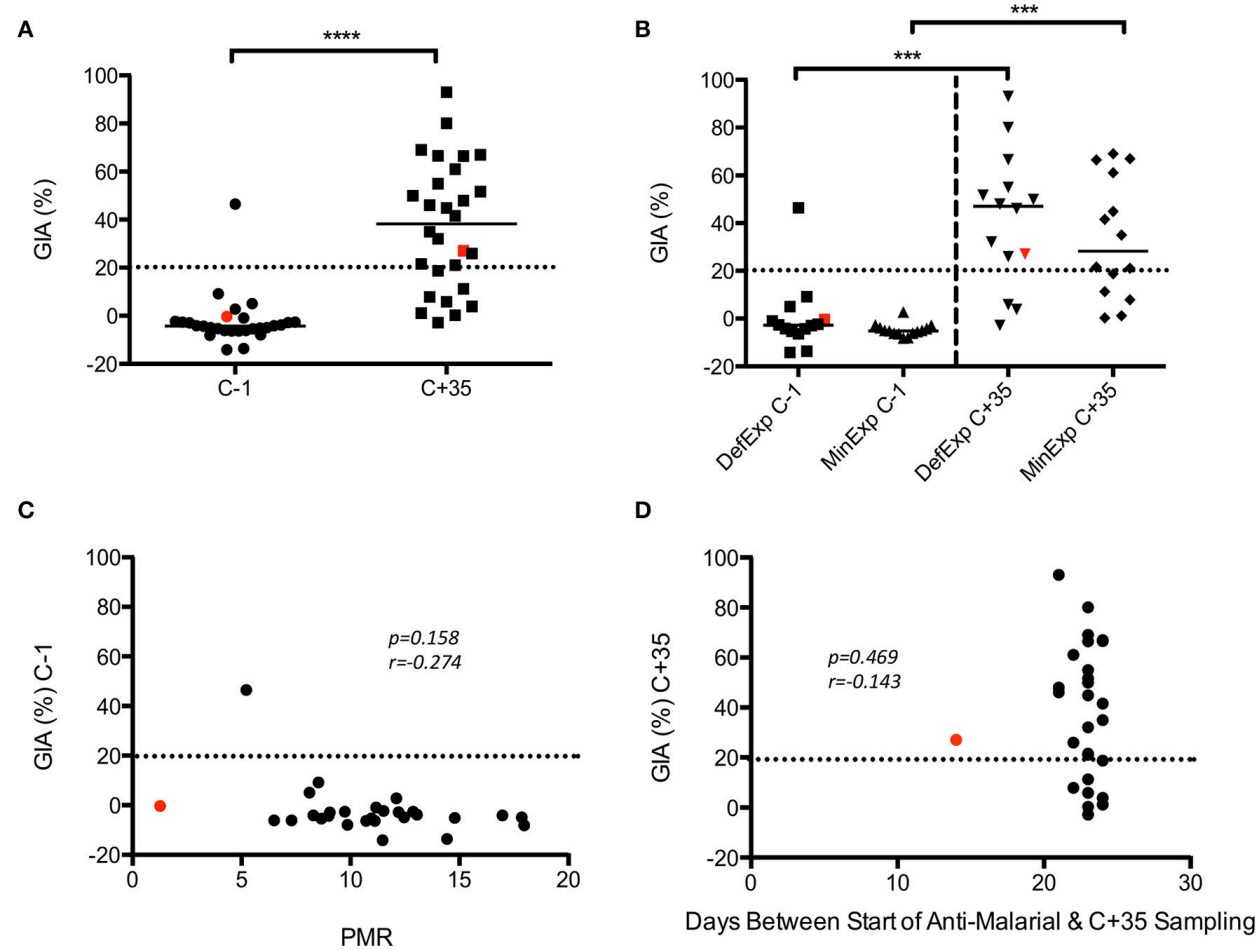
**KCS GIA. (A)** Pre- and post-CHMI. **(B)** MinExp and DefExp volunteers. **(C)** Correlation between GIA at C−1 and PMR. **(D)** Correlation between GIA at C+35 and days between start of anti-malaria therapy and sampling at C+35 visit. Data for volunteer 110 is highlighted in red. Individual and median values are indicated. Wilcoxon matched-pairs signed rank or Mann Whitney U tests as appropriate. Spearman rank test for correlations. MinExp, minimal prior exposure to malaria; DefExp, definite prior exposure to malaria. C−1 = baseline pre-CHMI. C+35 = 35 days post-CHMI. ^***^p < *0.001*, ^****^p < *0.0001*.

Analysis of GIA pre- and post-CHMI in KCS showed a significant increase post-infection (*p* ≤ *0.0001*) (Figure 4A). No significant difference in GIA was seen between MinExp and DefExp subjects at C−1 or C+35 (Figure 4B). Volunteer 110, who had a markedly reduced PMR *in vivo* post-infection (1.3/48 h; group median 11.1), demonstrated no GIA prior to CHMI (−0.4%) and only minimal GIA post-CHMI (27%). The subject with the highest GIA at C−1, in fact the only detectable response above background, volunteer 150 (46%) had DefExp to malaria and a PMR at the low end of the distribution seen across all the volunteers (5.2/48 h) (Figure S2). When all volunteers were included in the analysis, no correlation was seen between GIA and PMR or days between start of anti-malarial therapy and C+35 sampling (Figures 4C,D). However, the same anti-malarial therapy was used in both KCS and VAC049, and it is likely that GIA measured at C+35 in KCS was influenced to some degree by Atovaquone/Proguanil and so these results must be treated with caution.

### Analyses of ADRB activity

**Figure 5:**
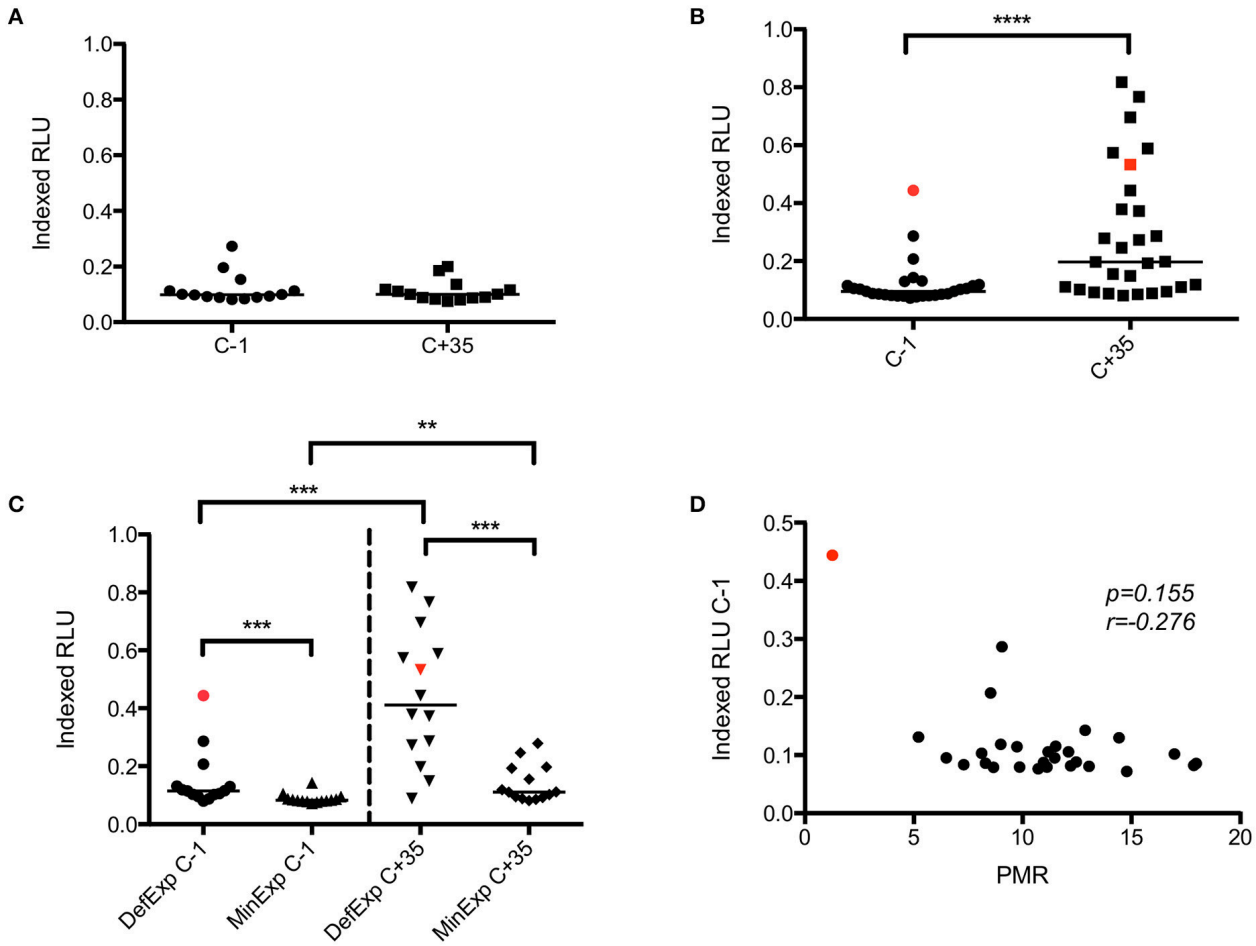
**VAC049 and KCS ADRB activity. (A)** VAC049 all infected volunteers pre- and post-CHMI. **(B)** KCS all volunteers pre- and post-CHMI. **(C)** KCS MinExp and DefExp volunteers. **(D)** KCS correlation between ADRB activity at C−1 and parasite multiplication rate (PMR). Data for volunteer 110 is highlighted in red. Individual and median values are indicated. RLU, relative light units. Wilcoxon matched-pairs signed rank or Mann Whitney U tests as appropriate. Spearman rank test for correlation. MinExp, minimal prior exposure to malaria; DefExp, definite prior exposure to malaria. C−1 = baseline pre-CHMI. C+35 = 35 days post-CHMI. ^**^p < *0.005*, ^***^p < *0.001*, ^****^p < *0.0001*.

In VAC049, no significant increase in ADRB activity was seen post-CHMI in infected volunteers (Figure 5A). Analysis of ADRB activity pre- and post-CHMI in KCS showed a significant increase post-infection (*p* ≤ *0.0001*) (Figure 5B). A significant difference in ADRB activity was seen between MinExp and DefExp subjects at both C−1 and C+35 (Figure 5C), consistent with the expected prior exposure status of these groups. Volunteer 110 had the highest ADRB at C−1, however, at 0.44 Indexed RLU this was considerably lower than the hyper-immune serum used as the positive control in the assay. Notably, this level of ADRB was maintained in this volunteer post-CHMI, and similar or higher levels were observed in roughly half of the volunteers in the DefExp group, also consistent with the observed increase in anti-malarial antibodies seen in the ELISA data (Figure 2). However, no overall correlation was seen between PMR and ADRB activity (Figure 5D). The ADRB assay at C−1 did not correlate with GIA, as has been reported recently in another study (Murungi et al., 2016).

## Discussion

We describe detailed serological and functional immunological responses for Kenyan and UK participants before and after CHMI with PfSPZ Challenge. This work is the first to compare immunological responses to a single episode of *P. falciparum* infection between malaria-naïve and exposed individuals and the first to allow correlation of these results with a measure of NAI—parasite multiplication rate *in vivo*.

Analysis of antibody responses to key blood-stage antigens revealed important information both about the malaria exposure of KCS volunteers and degree of exposure required for seroconversion. A single malaria infection was capable of inducing a significant increase in antibody responses against MSP1_19_, and AMA1 but not RH5 in malaria-naïve adults in VAC049, with response levels similar to those reported following mosquito-bite CHMI (Biswas et al., 2014). Of interest there was a significant difference in C+35 antibody responses to MSP1_19_, with a similar trend for AMA1, between VAC049 volunteers and MinExp subjects in KCS. These data support the presence of some pre-existing immunity to the AMA1 and MSP1_19_ antigens in this group of Kenyan adults, leading to stronger recall responses post-CHMI, likely attributable to memory B cell (mBC) responses induced by prior infections. Indeed, we have previously shown that a single CHMI exposure is sufficient to induce detectable levels of mBC against MSP1_19_ in UK adults (Elias et al., 2014), whilst mBC responses to both antigens are well maintained in Kenyan children who have experienced minimal prior exposure (Ndungu et al., 2012). For DefExp volunteers in KCS, only antibody responses at C+35 to MSP1_19_ and AMA1 were of a similar order to that reported for hyperimmune individuals (Biswas et al., 2014), supporting the suggestion that DefExp subjects in KCS likely had only mild to moderate prior exposure to malaria. In the KCS cohort, antibody responses to AMA1, and schizont at C−1 correlated with PMR, supporting published data showing a correlation between anti-AMA1 antibodies and risk of developing severe malaria in Kenyan children (Murungi et al., 2016), as well as the general notion that NAI is associated with an increasing magnitude and repertoire of anti-malarial antibody responses (Murungi et al., 2013; Rono et al., 2013) likely measured by schizont ELISA.

Development of a functional assay that could reliably predict NAI would have important applications, however validating candidate assays is extremely difficult. GIA and ADRB have both been suggested as candidate measures of NAI, however supporting evidence is limited (Joos et al., 2010; Duncan et al., 2012; Llewellyn et al., 2015). Although only one individual in KCS was qPCR positive but undiagnosed by C+21, analysis of PMRs showed a spread of values, allowing correlation of PMR with candidate *in vitro* assays.

In our study, the GIA assay measured the ability of purified IgG at 10 mg/mL to inhibit the growth of 3D7 clone blood-stage parasites *in vitro* over one life cycle. We used this clone as it is a genetic clone of the parental NF54 strain used for the PfSPZ Challenge. Volunteer 110 had undetectable GIA prior to CHMI and yet was able to control parasite growth *in vivo*. In contrast, subject 150 who had 46% GIA at C−1, was unable to control parasite growth to the same degree and was diagnosed with malaria following CHMI. These findings are in agreement with those of a recently published AMA1 vaccine study where despite induction of a median of 59.5% GIA (range 38.5–86.5%) using 10 mg/mL purified IgG (approximately the physiological level), no impact on PMR was seen following blood-stage CHMI (Payne et al., 2016). In contrast, non-human primate vaccine studies that have associated GIA *in vitro* with *in vivo* protection have achieved much higher levels of GIA, with protected animals showing >60% GIA when using 2.5 mg/mL IgG (roughly equivalent to a 1:4 serum dilution) (Singh et al., 2006; Douglas et al., 2015). These data suggest GIA induced by vaccines would represent a “non-natural” form of immunity, and such antibody-mediated protection requires a much higher threshold level than observed in clinical studies to date. Our results here suggest volunteer 110 was able to control parasite growth by a mechanism that does not inhibit merozoite invasion as measured by the GIA assay.

The finding of quinine in a UK volunteer's serum and the presumed effect of residual Atovaquone/Proguanil on GIA measurements at the C+35 time-point in VAC049 and KCS importantly suggest that anti-malarials, previously assumed to be removed by IgG purification with a Protein G column and subsequent dialysis, can give falsely high GIA results. Further work is needed to repeat this finding and investigate methods to ensure removal of anti-malarial medications from serum prior to the GIA assay. In the interim, caution should be taken interpreting GIA results from samples following anti-malarial therapy or where the use of anti-malarial drugs is unknown, for example in field studies. Investigators should also consider screening individuals demonstrating efficacy in CHMI trials for surreptitious anti-malarial use.

Whilst infected malaria-naïve volunteers in VAC049 failed to develop ADRB activity following a single malaria infection, both MinExp and DefExp subjects in KCS had a significant increase following CHMI, supporting the conclusion that MinExp volunteers had some degree of prior exposure to malaria. The increase in ADRB activity following CHMI was more marked in DefExp volunteers, possibly due to improved B cell memory or increased antibody levels capable of opsonizing merozoites in this group. Significant correlations were seen between ADRB activity at baseline and C+35 and antibody responses to MSP1_19_, AMA1 and schizont at these time points, further supporting the suggestion that ADRB activity correlates with exposure.

Whilst no correlation was seen between ADRB activity and parasitaemia at diagnosis or PMR, volunteer 110 did have the highest ADRB activity at baseline, suggesting the relationship between ADRB activity and NAI warrants further investigation. Interestingly, another study shows that volunteer 110 had titers of antibodies to the surface of infected erythrocytes (iRBC) that exceeded those of the hyperimmune positive control, suggesting this may be another possible mechanism by which PMR was controlled (*Abdi et al. Manuscript in preparation*). Delineating the overall contributions of anti-merozoite vs. anti-iRBC antibody responses to NAI (measured here by reduced *in vivo* PMR), will be an important focus of future research that should be greatly facilitated by access to the CHMI model in endemic areas.

Both VAC049 and KCS were pilot studies, with necessarily limited samples sizes. This, and the fact volunteers in KCS had only minimal to moderate NAI, meant the study was limited in its ability to test for associations between NAI and immunological readouts. That accepted, these findings, from hypothesis driven analyses, provide interesting results, suggesting that multiple antibody-dependent mechanisms are likely to contribute to protective immunity against malaria. In particular, the ADRB activity assay and antibodies against AMA1 and schizont extract show promise as measures of prior exposure to malaria and as possible predictors of control of parasite growth *in vivo*.

## Author contributions

Designed Studies: SHH, SD, SLH, BO, EJ, AH, KEM. Performed assays: SHH, DL, SS, KHM, SE, KAM, GK, CM, AM, JJ, AS, RL, TM. Analyzed Data: SHH, DL, SD, LD, CL, FO, Wrote manuscript: SHH, SD. Reviewed manuscript: All authors.

### Conflict of interest statement

SLH is an employee of Sanaria Inc. who manufactured PfSPZ Challenge. All the other authors declare that the research was conducted in the absence of any commercial or financial relationships that could be construed as a potential conflict of interest.

## Supplementary material

Figure S1 **Kaplan Meier plots of time to diagnosis in VAC049 and KCS. (A)** VAC049 where median pre-patent period = 13.19 days for 2500 SPZ ID; 17.8 days for 2500 SPZ IM; and 12.72 days for 25,000 SPZ IM. Comparison between all groups: *p* = 0.024, Log rank test. **(B)** KCS where median pre-patient period = 12.2 days for minimally exposed and 12.1 days for definitely exposed. **(C)** VAC049 and KCS showing diagnosed volunteers only (i.e., excluding volunteer 110). *P*-values = log rank tests. Days post-administration of PfSPZ Challenge = day between injection and diagnosis. ID, intradermal; IM, intramuscular. MinExp, Volunteers with minimal prior exposure to malaria. DefExp, Definite prior exposure to malaria. These data have been reported previously (Hodgson et al., 2014) but are replotted here for completeness.

Figure S2 **Parasite multiplication rates for all volunteers in KCS**. 95% Confidence intervals for each value are indicated. PMR, parasite multiplication rate (fold change in parasites over 48 h). Volunteer 110 is highlighted in red. These data have been reported previously (Hodgson et al., 2014) but are replotted here for completeness.

Figure S3 **Comparison of Liver to Blood inoculum between volunteers diagnosed with malaria in KCS and VAC049**. LBI, liver to blood inoculum—total number of parasites released from liver on C+6.5 as modeled from the qPCR data.

Figure S4 **Serum IgG antibody responses pre- and post-CHMI for VAC049 and volunteers with minimal prior exposure to malaria in KCS. (A)** VAC049 (*n* = 14). Responses are shown before (C−1) and after (C+35) CHMI. **(B)** Antibody responses at C−1 for VAC049 and volunteers with minimal prior exposure to malaria (MinExp) in KCS (*n* = 14). **(C)** Antibody responses at C+35 for VAC049 and MinExp volunteers from KCS. Median and individual values are indicated. Comparisons were performed using Wilcoxon matched-pairs signed rank or Mann Whitney U tests as appropriate. It should be noted that each ELISA assay reports antibody responses in arbitrary units, and the magnitude of these cannot be compared between antigens. ^**^*p* < 0.005, ^***^*p* < 0.001.

Table S1 **Analysis of antibody responses, GIA and ADRB activity for VAC049: Only subjects infected in VAC049 were included (*n* = 14)**. GIA was not available for VAC049 volunteer 1224 at C−1. ELISA responses for RH5 were not included in the table as most values were negative. Correlations were performed using Spearman rank test. Comparisons were performed using Wilcoxon matched-pairs signed rank or Mann Whitney U tests as appropriate. ^*^GIA data not reliable due to likely persistence of anti-malarial drug.
