# Supplementary figures and images for: Changes in Serological Immunology Measures in UK and Kenyan Adults Post-controlled Human Malaria Infection
Source: Front Microbiol. 2016 Oct 13;7:1604. doi: 10.3389/fmicb.2016.01604 (PMC5061779; doi:10.3389/fmicb.2016.01604)

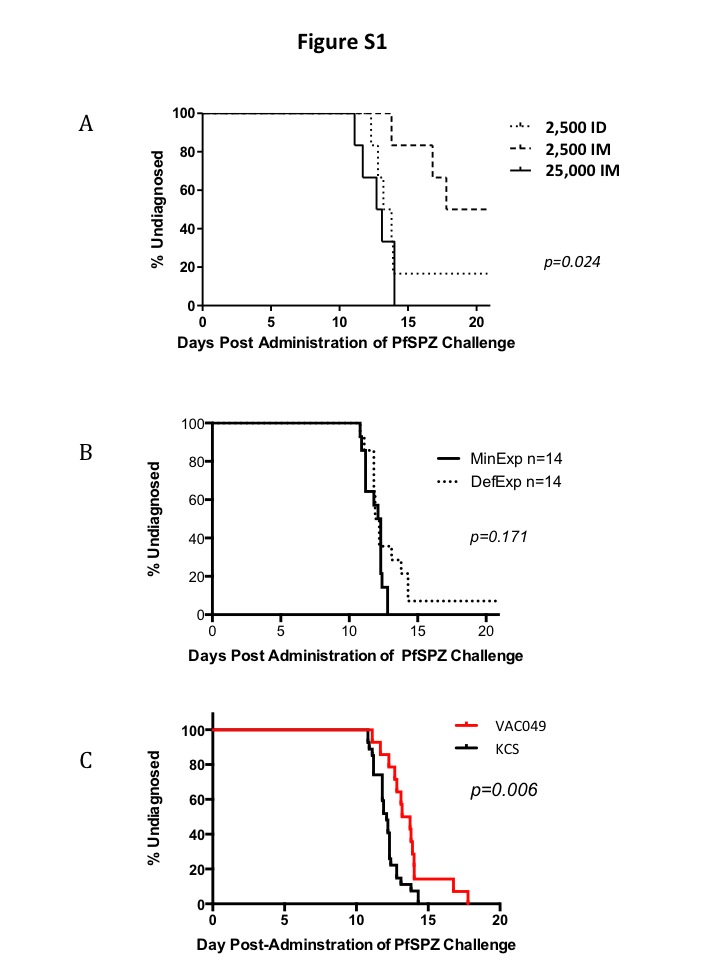

Supplement: Figure S1 — Kaplan Meier plots of time to diagnosis in VAC049 and KCS. (A) VAC049 where median pre-patent period = 13.19 days for 2500 SPZ ID; 17.8 days for 2500 SPZ IM; and 12.72 days for 25,000 SPZ IM. Comparison between all groups: p = 0.024, Log rank test. (B) KCS where median pre-patient period = 12.2 days for minimally exposed and 12.1 days for definitely exposed. (C) VAC049 and KCS showing diagnosed volunteers only (i.e., excluding volunteer 110). P-values = log rank tests. Days post-administration of PfSPZ Challenge = day between injection and diagnosis. ID, intradermal; IM, intramuscular. MinExp, Volunteers with minimal prior exposure to malaria. DefExp, Definite prior exposure to malaria. These data have been reported previously (Hodgson et al., 2014) but are replotted here for completeness. [file Image1.jpeg]

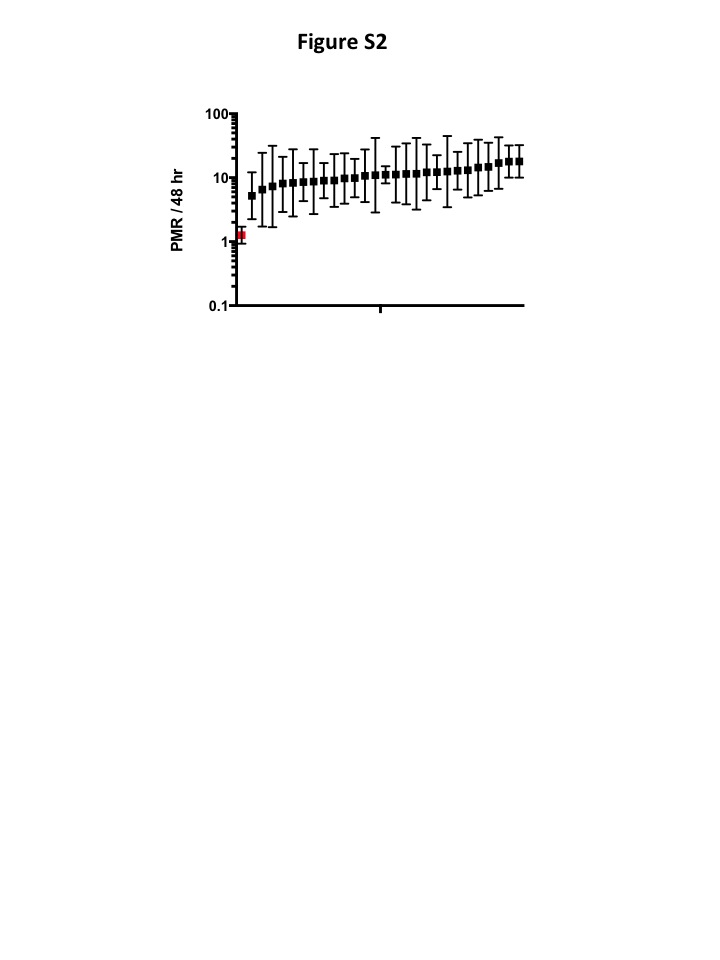

Supplement: Figure S2 — Parasite multiplication rates for all volunteers in KCS. 95% Confidence intervals for each value are indicated. PMR, parasite multiplication rate (fold change in parasites over 48 h). Volunteer 110 is highlighted in red. These data have been reported previously (Hodgson et al., 2014) but are replotted here for completeness. [file Image2.jpeg]

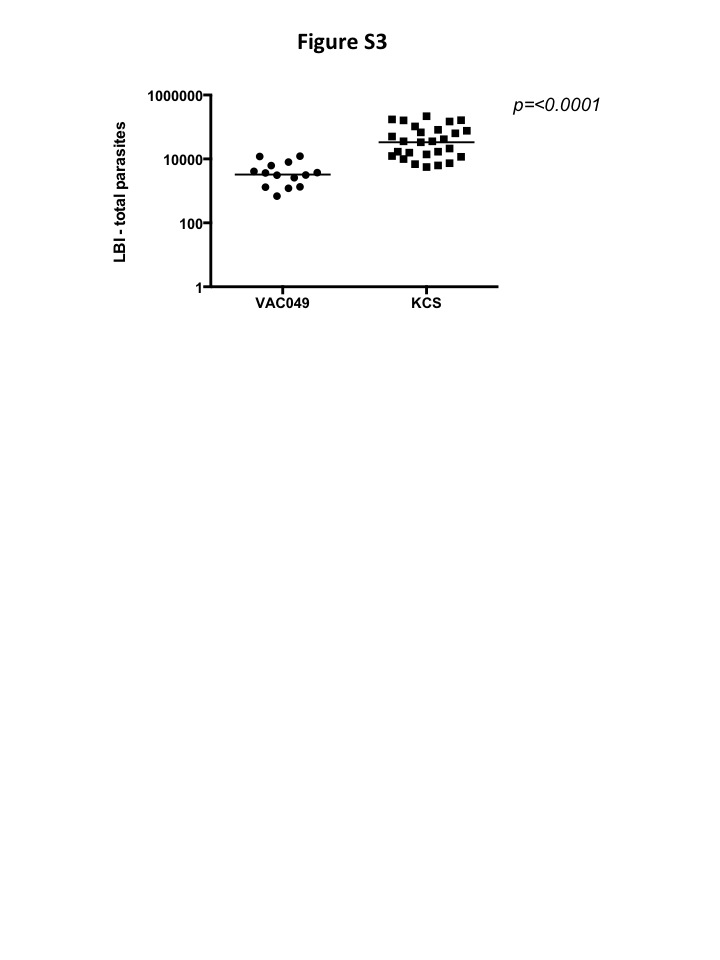

Supplement: Figure S3 — Comparison of Liver to Blood inoculum between volunteers diagnosed with malaria in KCS and VAC049. LBI, liver to blood inoculum—total number of parasites released from liver on C+6.5 as modeled from the qPCR data. [file Image3.jpeg]

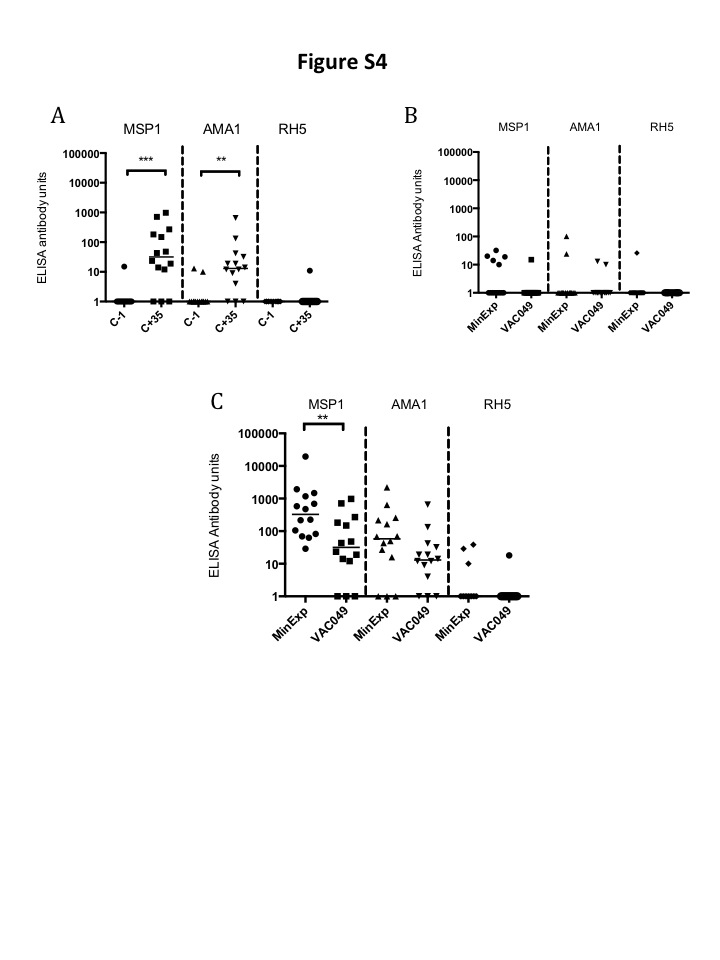

Supplement: Figure S4 — Serum IgG antibody responses pre- and post-CHMI for VAC049 and volunteers with minimal prior exposure to malaria in KCS. (A) VAC049 (n = 14). Responses are shown before (C−1) and after (C+35) CHMI. (B) Antibody responses at C−1 for VAC049 and volunteers with minimal prior exposure to malaria (MinExp) in KCS (n = 14). (C) Antibody responses at C+35 for VAC049 and MinExp volunteers from KCS. Median and individual values are indicated. Comparisons were performed using Wilcoxon matched-pairs signed rank or Mann Whitney U tests as appropriate. It should be noted that each ELISA assay reports antibody responses in arbitrary units, and the magnitude of these cannot be compared between antigens. **p < 0.005, ***p < 0.001. [file Image4.jpeg]
