# Supplementary material for: Changes in Serological Immunology Measures in UK and Kenyan Adults Post-controlled Human Malaria Infection
Source: Front Microbiol. 2016 Oct 13;7:1604. doi: 10.3389/fmicb.2016.01604 (PMC5061779; doi:10.3389/fmicb.2016.01604)
Supplement: Table S1 — Analysis of antibody responses, GIA and ADRB activity for VAC049: Only subjects infected in VAC049 were included (n = 14). GIA was not available for VAC049 volunteer 1224 at C−1. ELISA responses for RH5 were not included in the table as most values were negative. Correlations were performed using Spearman rank test. Comparisons were performed using Wilcoxon matched-pairs signed rank or Mann Whitney U tests as appropriate. *GIA data not reliable due to likely persistence of anti-malarial drug. [file DataSheet1.DOCX]

**SUPPLEMENTARY INFORMATION**

**Clinical Trial Ethical & Regulatory Approval**

*KCS:* Ethical approval was granted by the ERC (KEMRI/RES/7/3/1) and the Oxford Tropical Research Ethics Committee (OXTREC 161-12). Regulatory approval was provided by the Kenyan Pharmacy and Poisons Board (PPB/ECCT/12/12/01/2013). The study was reviewed and allowed to proceed by the US Food and Drug Administration under IND 14267. The Safety Monitoring Committee (SMC) provided safety oversight and GCP compliance was independently monitored by the Clinical Trials Facility, Monitoring Department, Kenya Medical Research Institute and Wellcome Trust (Centre for Geographical Medicine Research – Coast), Kilifi, Kenya.

*VAC049:* Ethical approval was granted by the UK National Research Ethics Committee South Central Oxford A (Ref: 11/SC/0351). UK regulatory approval of the trial was not necessary as PfSPZ Challenge is considered a non-investigational product in the UK. The study was reviewed and allowed to proceed by the US Food and Drug Administration under IND 14267. The trial was registered with ClinicalTrials.gov (NCT01465048). The Local Safety Committee provided safety oversight and GCP compliance was independently monitored by the University of Oxford’s Department for Clinical Trials and Research Governance.

**qPCR and Parasite Growth Modelling**

For modelling purposes, in the following circumstances the qPCR value was replaced with the nominal value of 5 p/ml:

- Any qPCR negative time-points immediately followed by a qPCR positive time-point.
- qPCR values <5/ml but > 0 p/mL.

Time in hours between CHMI and collection of each blood-sample was calculated using data specific to each volunteer for each of these events and then converted to days. Simple linear regression([Douglas et al., 2013](#_ENREF_1)) was then used to estimate;

1. Parasite multiplication rate (PMR): The fold change in number of parasites in the blood over 48 hours. PMR was only calculated for volunteers with 5 or more positive qPCR values post-CHMI.

$$PMR=10^(m\times2)$$

1. Liver to blood inoculum (LBI): The total number of parasites released from the liver into the blood at C+6.5 post-CHMI (BV = blood volume; 70mL per kg).

$$LBI=\left( m\times6.5 \right)-y)\left( BV \right)$$

##### **Anti-AMA1, Anti-MSP1 and Anti-RH5 ELISAs**

Sera from participants were analysed for antibody responses according to published standardized ELISA methodology([Miura et al., 2008](#_ENREF_7);[Sheehy et al., 2011](#_ENREF_10)). Recombinant ETSR-MSP1_19_ fusion protein (3D7 allelle)([Goodman et al., 2010](#_ENREF_2)) 3D7 AMA1 protein([Sheehy et al., 2012](#_ENREF_9)) and RH5 protein were diluted in PBS to a previously optimised concentration (2 μg/mL). The RH5 protein encoded the full-length ectodomain of 3D7 RH5 (amino acids E26-Q526), where all four putative N-linked glycosylation sequons (N-X-S/T) were mutated Thr to Ala ([Hjerrild et al., 2016](#_ENREF_3)). The C-terminus included a ‘C-tag’ – four amino acids E-P-E-A used to purify the protein. The protein was secreted into and purified from the supernatant of a stable *Drosophila* S2 stable cell line encoding the RH5 construct [Jin *et al.*, in preparation]. 50 μL protein/well was adsorbed to 96-well Nunc-Immuno Maxisorp plates (Fisher Scientific) and stored at 4 **°**C for approximately 16 h. Plates were washed 6x in PBS/T and blocked with Caesin blocker (Pierce) for at least 1 h at room temperature (RT). Test sera were diluted in PBS/T (1:300, 1:3,000 and 1:30,000) and added to wells in duplicate.

Sera from hyperimmune adults were used as positive controls and to make standard curves for the MSP1 and AMA1 ELISAs. Sera from a malaria-naïve volunteer vaccinated with viral vectored vaccines encoding RH5 (NCT02181088) was used as a positive control and to make the standard curve for the RH5 ELISA. For the standard curves, sera were diluted in PBS (Pierce) to 1:900 for MSP1_19_, ELISAs 1:1000 for AMA1 ELISAs and 1:100 for RH5 ELISAs. Sera for positive controls was diluted 1:3600 for MSP1 ELISAs, 1:8000 for AMA1 ELISAs and 1:3200 for RH5 ELISAs.

Plates were incubated for 2 hours at RT, after which they were washed 6x with PBS/T. Plates were incubated with secondary antibody (50 μL/well of goat anti-human total IgG (γ-chain)(Sigma A3187) diluted 1:1000 in Caesin blocker) for 1 h at RT. Plates were washed 6x with PBS/T and 100 μL of DB added to each well.

Optical density was read at 405nm (OD_405_). Plates were read after 5 min incubation in the dark at RT or until positive control wells gave an expected OD_405_.

##### **Anti-schizont extract ELISA**

Individual wells of Dynex Immunolon 4HBX ELISA plates (Dynex Technologies Inc) were coated with *P. falciparum* schizont extract (A4 and 3D7 parasite strains) was coated onto wells in PBS([Ndungu et al., 2002](#_ENREF_8)). Plates were incubated overnight at 4 ^o^C, before washing four times in PBS/T, and blocking for 5 h at RT with 1% skimmed milk in PBS/T (blocking buffer). Wells were washed again and incubated overnight at 4 °C with 100 μL of test sera (1:1000 dilution in blocking buffer). Plates were then washed 4x and incubated for 3 h at RT with 100 μL of HRP-conjugated rabbit anti-human IgG, (Dako Ltd.) at 1:5000 dilution in blocking buffer before final washing and detection with H_2_O_2_ and O-phenylenediamine (Sigma). The reaction was stopped with 25 μL of 2 M H_2_SO_4_ per well and absorbance read at 492 nm. The same positive controls (hyper-immune sera) were run in duplicate on each plate to allow for standardization of plate-to-plate variation. As high background reactivity was seen with non-malaria exposed sera (or UK sera) in results using 3D7 lysate, final results were obtained using A4 lysates (ELISA ODs to A4 and 3D7 lysates were highly correlated, data not shown).

##### **Growth inhibition activity assay**

The ability of antibodies to inhibit growth of *P. falciparum* 3D7 clone parasites *in vitro* was assessed by a standardized GIA assay using purified IgG as previously described([Malkin et al., 2005](#_ENREF_6)). This assay was performed at the GIA Reference Center (Laboratory of Malaria and Vector Research, NIH). Serum from participants in VAC049 and KCS were tested against 3D7 parasites at 10 mg/ml. IgG was purified from serum using protein G columns and purified polyclonal IgG samples were dialysed and concentrated. Concentrated IgG was pre-absorbed with uninfected human O+ RBCs for 1 h. *P. falciparum* late trophozoites and schizonts were mixed with test or control samples and culture medium in 96-well culture plates. The final concentration of the culture was 0.3 ± 0.1% parasitaemia, 1% hematocrit in growth medium. Each sample was tested in triplicate. Cultures were maintained for a single growth cycle (40-42 h) and relative parasitemia quantified by biochemical determination of parasite lactate dehydrogenase.

##### **Antibody-dependent respiratory burst assay**

Detailed metholdology can be found in the recent publication by Llewellyn et al([Llewellyn et al., 2015](#_ENREF_5)). Briefly, three sets of *P. falciparum* Mz lysate were produced and parasitophorous vacuolar membrane (PVM)-enclosed merozoite structures (PEMS) harvested and re-suspended at a concentration of 18.5 x 10^5^ schizonts/mL in PBS. Human polymorphonuclear neutrophils (PMNs) were prepared fresh for each assay and used within 3 hours of collection. 100 μL *P. falciparum* PEMS at 18.5 x 10^5^ schizonts/mL was adsorbed onto Nunc opaque plates which were then blocked for 1 h with Casein block solution (Pierce). 100μL serum diluted 1:50 in PBS was then added and incubated for 1 h at 37 °C. Within 2 min of a final wash of the assay plate in PBS, 50 μL isoluminol (0.04 mg/mL) (Sigma) and 50 μL of isolated human PMNs at 1 x 10^7^ PMNs/mL were added to each well and luminescence was read every 2 min for 1 h.

Every sample was tested with a 3 different neutrophil donors. Maximum relative light units (RLU) were recorded and indexed against the hyperimmune positive control (‘reference’) maximum RLU as below:

Indexed RLU $= \frac{Absolute maximum RLU of test sample}{Absolute maximum RLU of reference}$

After indexing, the mean of the three replicates for each sample was reported.

**Testing for Anti-Malarial Compounds in Serum**

Serum from volunteer 1221 in VAC49 that demonstrated 99.9% GIA prior to CHMI was screened for the presence of 14 anti-malarial medications (Piperaquine, Amodiaquine, Desethyl-amodiaquine, Chloroquine, Pyronaridine, Quinine, Sulfadoxine, Pyrimethamine, Mefloquine, Artesunate, Artemether, Dihydroartemisinin, Lumefantrine and Desbutyl-lumefantrine) with an adaption of the previously published liquid chromatography-tandem mass spectrometry assay using stable isotopically labeled internal standards for quantification([Hodel et al., 2009](#_ENREF_4)).

**SUPPLEMENTARY TABLES**

Table S1: Analysis of antibody responses, GIA and ADRB activity for VAC049: Only subjects infected in VAC049 were included (n=14). GIA was not available for VAC049 volunteer 1224 at C-1. ELISA responses for RH5 were not included in the table as most values were negative. Correlations were performed using Spearman rank test. Comparisons were performed using Wilcoxon matched-pairs signed rank or Mann Whitney U tests as appropriate. *=GIA data not reliable due to likely persistence of anti-malarial drug.

**SUPPLEMENTARY REFERENCES**

Douglas, A.D., Edwards, N.J., Duncan, C.J., Thompson, F.M., Sheehy, S.H., O'hara, G.A., Anagnostou, N., Walther, M., Webster, D.P., Dunachie, S.J., Porter, D.W., Andrews, L., Gilbert, S.C., Draper, S.J., Hill, A.V., and Bejon, P. (2013). Comparison of Modeling Methods to Determine Liver-to-blood Inocula and Parasite Multiplication Rates During Controlled Human Malaria Infection. *J Infect Dis*.

Goodman, A.L., Epp, C., Moss, D., Holder, A.A., Wilson, J.M., Gao, G.P., Long, C.A., Remarque, E.J., Thomas, A.W., Ammendola, V., Colloca, S., Dicks, M.D., Biswas, S., Seibel, D., Van Duivenvoorde, L.M., Gilbert, S.C., Hill, A.V., and Draper, S.J. (2010). New candidate vaccines against blood-stage Plasmodium falciparum malaria: prime-boost immunization regimens incorporating human and simian adenoviral vectors and poxviral vectors expressing an optimized antigen based on merozoite surface protein 1. *Infect Immun* 78**,** 4601-4612.

Hjerrild, K.A., Jin, J., Wright, K.E., Brown, R.E., Marshall, J.M., Labbe, G.M., Silk, S.E., Cherry, C.J., Clemmensen, S.B., Jorgensen, T., Illingworth, J.J., Alanine, D.G., Milne, K.H., Ashfield, R., De Jongh, W.A., Douglas, A.D., Higgins, M.K., and Draper, S.J. (2016). Production of full-length soluble Plasmodium falciparum RH5 protein vaccine using a Drosophila melanogaster Schneider 2 stable cell line system. *Sci Rep* 6**,** 30357.

Hodel, E.M., Zanolari, B., Mercier, T., Biollaz, J., Keiser, J., Olliaro, P., Genton, B., and Decosterd, L.A. (2009). A single LC-tandem mass spectrometry method for the simultaneous determination of 14 antimalarial drugs and their metabolites in human plasma. *J Chromatogr B Analyt Technol Biomed Life Sci* 877**,** 867-886.

Llewellyn, D., Miura, K., Fay, M.P., Williams, A.R., Murungi, L.M., Shi, J., Hodgson, S.H., Douglas, A.D., Osier, F.H., Fairhurst, R.M., Diakite, M., Pleass, R.J., Long, C.A., and Draper, S.J. (2015). Standardization of the antibody-dependent respiratory burst assay with human neutrophils and Plasmodium falciparum malaria. *Sci Rep* 5**,** 14081.

Malkin, E.M., Diemert, D.J., Mcarthur, J.H., Perreault, J.R., Miles, A.P., Giersing, B.K., Mullen, G.E., Orcutt, A., Muratova, O., Awkal, M., Zhou, H., Wang, J., Stowers, A., Long, C.A., Mahanty, S., Miller, L.H., Saul, A., and Durbin, A.P. (2005). Phase 1 clinical trial of apical membrane antigen 1: an asexual blood-stage vaccine for Plasmodium falciparum malaria. *Infect Immun* 73**,** 3677-3685.

Miura, K., Orcutt, A.C., Muratova, O.V., Miller, L.H., Saul, A., and Long, C.A. (2008). Development and characterization of a standardized ELISA including a reference serum on each plate to detect antibodies induced by experimental malaria vaccines. *Vaccine* 26**,** 193-200.

Ndungu, F.M., Bull, P.C., Ross, A., Lowe, B.S., Kabiru, E., and Marsh, K. (2002). Naturally acquired immunoglobulin (Ig)G subclass antibodies to crude asexual Plasmodium falciparum lysates: evidence for association with protection for IgG1 and disease for IgG2. *Parasite Immunol* 24**,** 77-82.

Sheehy, S.H., Duncan, C.J., Elias, S.C., Choudhary, P., Biswas, S., Halstead, F.D., Collins, K.A., Edwards, N.J., Douglas, A.D., Anagnostou, N.A., Ewer, K.J., Havelock, T., Mahungu, T., Bliss, C.M., Miura, K., Poulton, I.D., Lillie, P.J., Antrobus, R.D., Berrie, E., Moyle, S., Gantlett, K., Colloca, S., Cortese, R., Long, C.A., Sinden, R.E., Gilbert, S.C., Lawrie, A.M., Doherty, T., Faust, S.N., Nicosia, A., Hill, A.V., and Draper, S.J. (2012). ChAd63-MVA-vectored blood-stage malaria vaccines targeting MSP1 and AMA1: assessment of efficacy against mosquito bite challenge in humans. *Mol Ther* 20**,** 2355-2368.

Sheehy, S.H., Duncan, C.J., Elias, S.C., Collins, K.A., Ewer, K.J., Spencer, A.J., Williams, A.R., Halstead, F.D., Moretz, S.E., Miura, K., Epp, C., Dicks, M.D., Poulton, I.D., Lawrie, A.M., Berrie, E., Moyle, S., Long, C.A., Colloca, S., Cortese, R., Gilbert, S.C., Nicosia, A., Hill, A.V., and Draper, S.J. (2011). Phase Ia clinical evaluation of the Plasmodium falciparum blood-stage antigen MSP1 in ChAd63 and MVA vaccine vectors. *Mol Ther* 19**,** 2269-2276.
